# Supplementary figures and images for: Biochemical properties of naturally occurring human bloom helicase variants
Source: PLoS One. 2023 Jun 2;18(6):e0281524. doi: 10.1371/journal.pone.0281524 (PMC10237670; doi:10.1371/journal.pone.0281524)

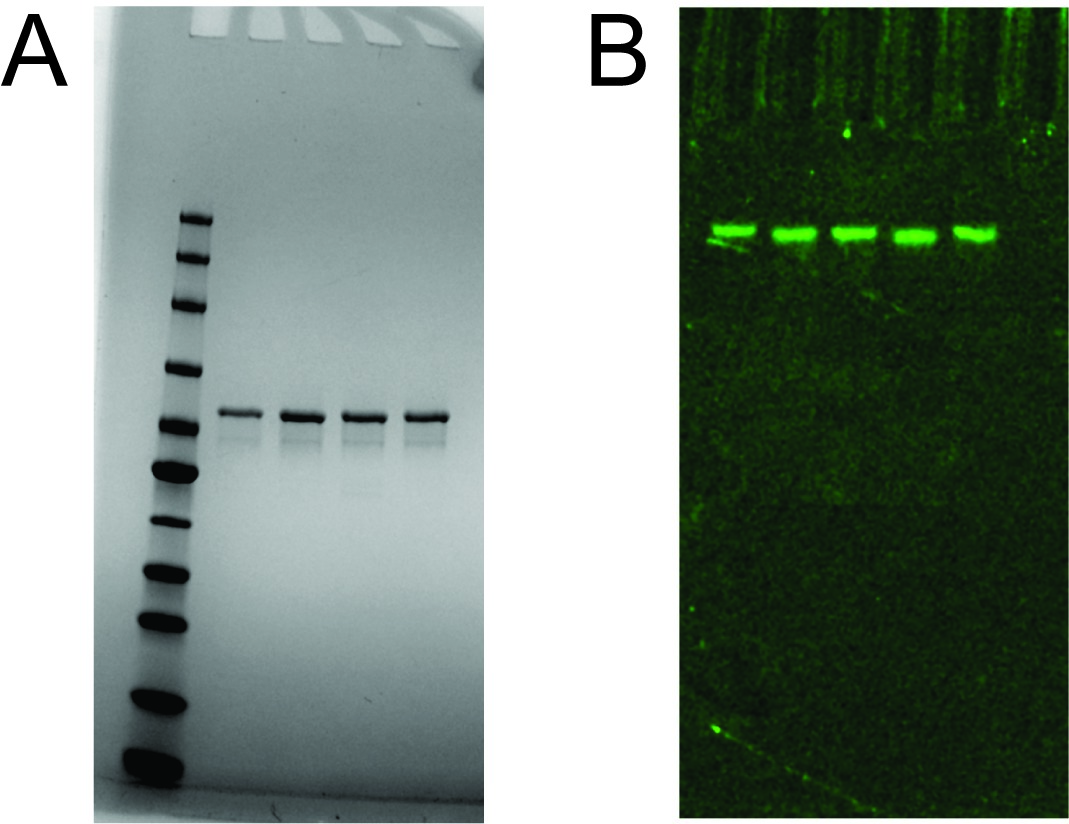

Supplement: S1 Fig — (A). Assessment of the purity of WT BLMcore and BLMcore variants. Left to right: Blue Prestained Broad Range Protein Standard (New England Biolabs), WT BLMcore, BLMcore P868L, BLMcore G1120R, and BLMcore K869A K870A loaded onto a 4–15% Mini-PROTEAN TGX Precast Protein gel (BioRad). Each protein was at a concentration of ~2.5 μM. (B). Assessment of nuclease contamination of WT BLMcore and BLMcore variants at 1 μM concentration. Proteins were incubated with 40 nM fluorescein labeled dT30 in 50 mM Tris-HCl, pH 7.5, 50 mM KCl, 1 mM DTT, 0.1 mg/mL Bovine Serum Albumin (BSA), and 5 mM MgCl2 for 30 minutes at room temperature. Five μL of stop buffer (2% SDS, 5 μg/mL proteinase K, 20% (v/v) glycerol, 0.1 ethylenediaminetetraacetic acid (EDTA)) was added to each sample, and 5 μL of each sample was loaded onto a 15% acrylamide 1.5-mm gel in Tris-Borate-EDTA (TBE) buffer supplemented with 100 mM KCl. Gels were run at 75 V for 1 hour at 4°C in 1xTBE running buffer with 100 mM KCl and imaged on an Azure c600 (Azure Biosystems). Samples in gel (from left to right) are DNA alone, WT BLM, BLM P868L, BLM G1120R, and BLM K869A K870A. (TIF) [file pone.0281524.s001.tif]

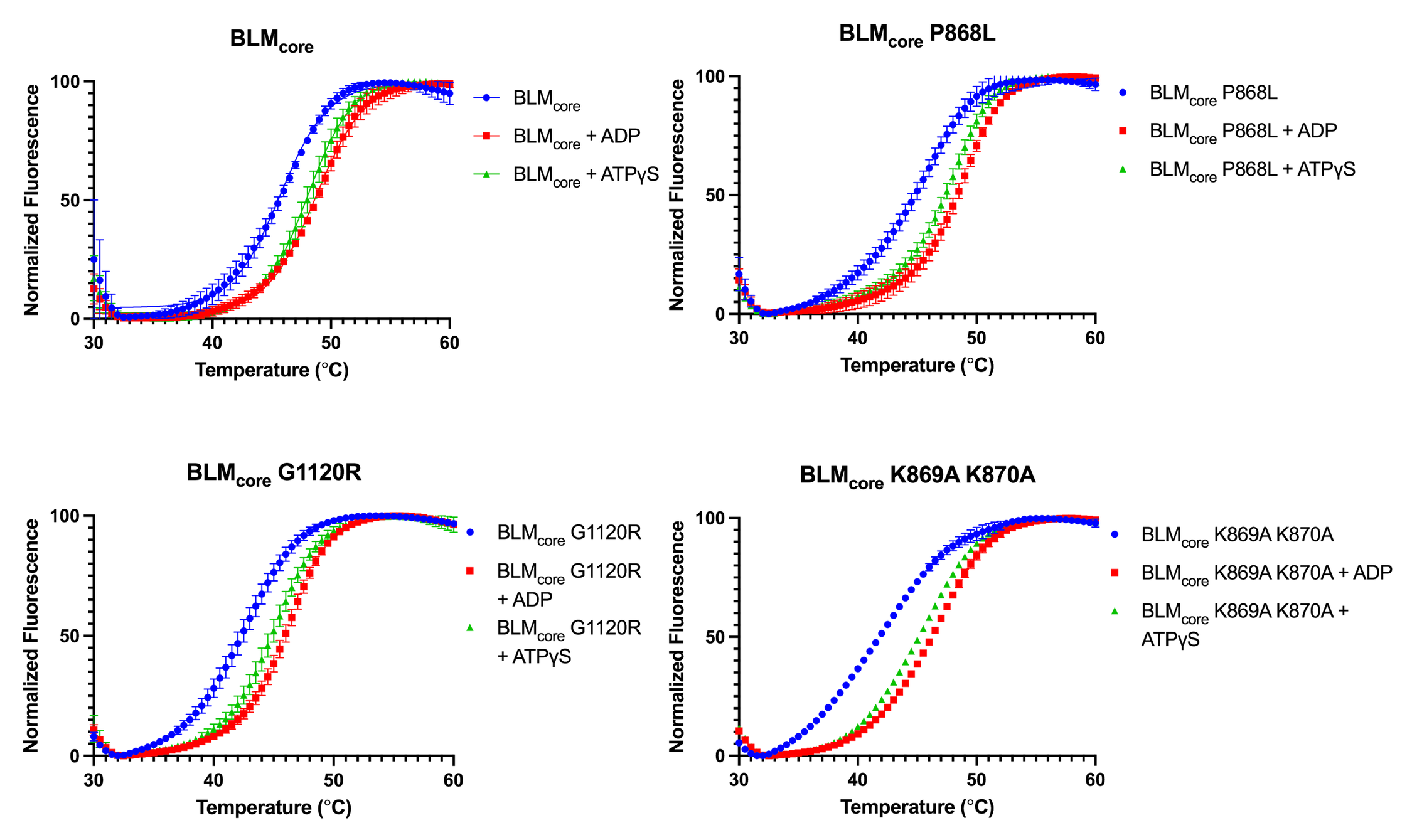

Supplement: S2 Fig — The normalized melt curves between 30°C and 60°C is shown for BLMcore, BLMcore P868L, BLMcore G1120R, and BLMcore K869A K870A in the absence (blue circles) or presence of 0.5 mM ADP (red squares) or ATPγS (green triangle). Points on graph indicate the mean of three replicates and error bars indicate standard deviation calculated by Prism (version 9.3.1). (TIF) [file pone.0281524.s002.tif]

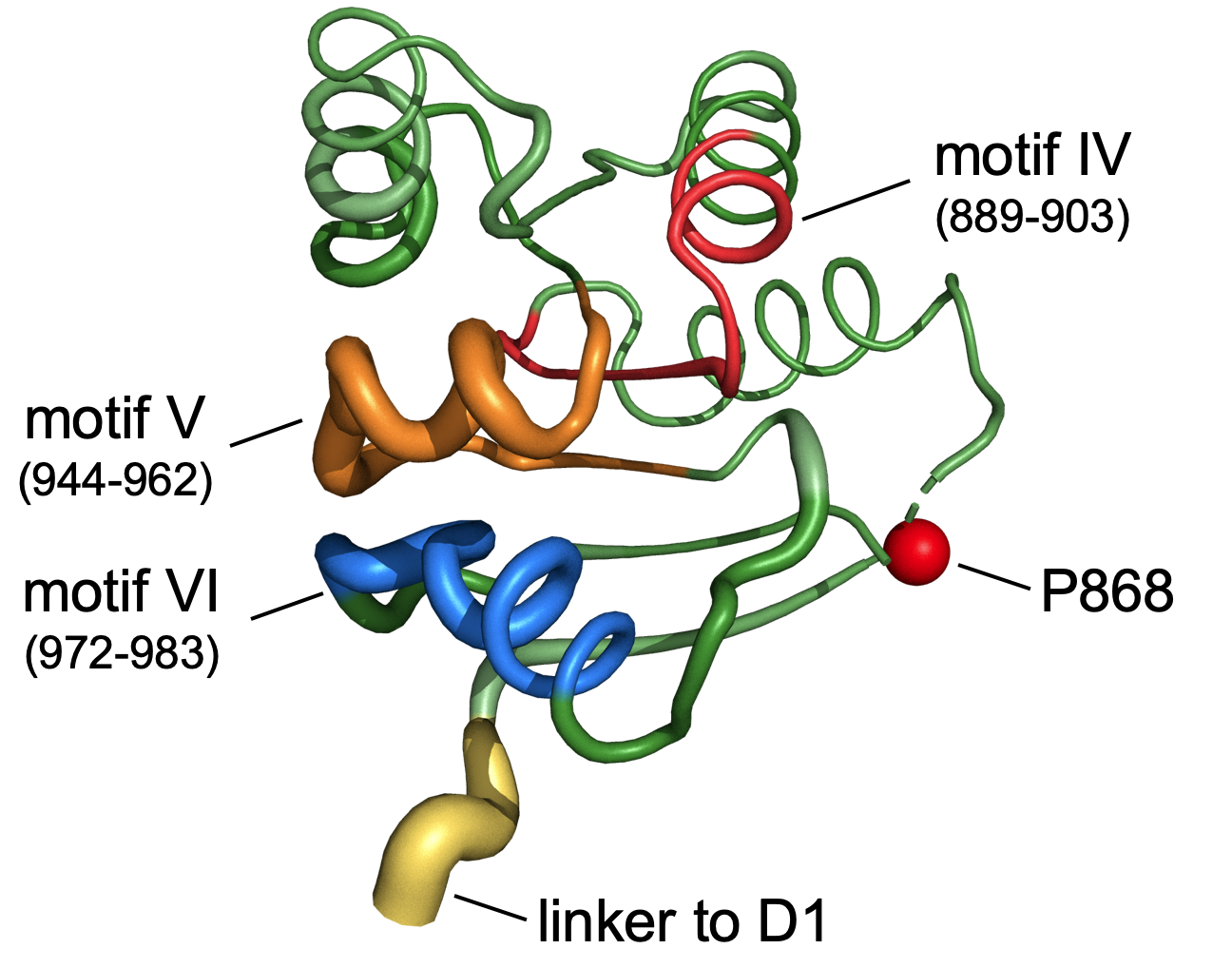

Supplement: S3 Fig — The X-ray structure of the D2 domain is shown as a cartoon putty where the width of the putty denotes the magnitude of η. The three conserved helicase motifs present in D2 and the linker connecting D2 to D1 are highlighted in color. The red sphere indicates the position of the P868 residue at the opposite side of D2. (TIF) [file pone.0281524.s003.tif]
