# Supplementary material for: Biochemical properties of naturally occurring human bloom helicase variants
Source: PLoS One. 2023 Jun 2;18(6):e0281524. doi: 10.1371/journal.pone.0281524 (PMC10237670; doi:10.1371/journal.pone.0281524)
Supplement: Attachment — Submitted filename: Response to Reviewers - incorporating KS edits.docx [file pone.0281524.s004.docx]

Reviewers' comments:

Reviewer's Responses to Questions

**Comments to the Author**

1. Is the manuscript technically sound, and do the data support the conclusions?

The manuscript must describe a technically sound piece of scientific research with data that supports the conclusions. Experiments must have been conducted rigorously, with appropriate controls, replication, and sample sizes. The conclusions must be drawn appropriately based on the data presented.

Reviewer #1: Partly

Reviewer #2: Yes

2. Has the statistical analysis been performed appropriately and rigorously?

Reviewer #1: Yes

Reviewer #2: Yes

3. Have the authors made all data underlying the findings in their manuscript fully available?

The [PLOS Data policy](http://www.plosone.org/static/policies.action#sharing) requires authors to make all data underlying the findings described in their manuscript fully available without restriction, with rare exception (please refer to the Data Availability Statement in the manuscript PDF file). The data should be provided as part of the manuscript or its supporting information, or deposited to a public repository. For example, in addition to summary statistics, the data points behind means, medians and variance measures should be available. If there are restrictions on publicly sharing data—e.g. participant privacy or use of data from a third party—those must be specified.

Reviewer #1: Yes

Reviewer #2: Yes

4. Is the manuscript presented in an intelligible fashion and written in standard English?

PLOS ONE does not copyedit accepted manuscripts, so the language in submitted articles must be clear, correct, and unambiguous. Any typographical or grammatical errors should be corrected at revision, so please note any specific errors here.

Reviewer #1: Yes

Reviewer #2: Yes

5. Review Comments to the Author

Please use the space provided to explain your answers to the questions above. You may also include additional comments for the author, including concerns about dual publication, research ethics, or publication ethics. (Please upload your review as an attachment if it exceeds 20,000 characters)

Reviewer #1: Biochemical Properties of Naturally Occurring Human Bloom Helicase Variants

The authors present a set of biochemical experiments that set out to test the effects of two single nucleotide polymorphisms on the in vitro activities of recombinant human BLM helicase; including unwinding of different DNA substrates, EMSAs, ATPase assays and molecular dynamics calculations.

MAJOR POINTS

Page 6, Line 125 – ‘We also measured activity of a BLMcore K869A K870A mutant protein to assess the role of the lysines within the lysine-rich loop’.
>> Examination of the indicated PDB deposition, indicates that the sidechain of Lys870 is not involved in any polar interactions with the bound substrate (but provides a van der Waals surface), whereas Lys869 is involved in a complex series of internal stabilising hydrogen bonds, including the sidechain and backbone of Ser965, and the sidechain of Asp997. It is therefore not clear why mutations in these residues were chosen for examination in this study. Lys872 (and perhaps 873) due to the proximity to the phosphodiester backbone may have been more rational choices.

We examined the lysine-rich loop because of the negative impacts of BLM P868L in cells. Our goal was to investigate whether the lysine-rich loop that residue P868 is part of is generally important for BLM structure and function and residues K869 K870 due to their proximity to P868. We reasoned that this mutant construct could reveal possible roles for the lysine-rich loop that P868 is embedded within.

Page 8, Line 178 — ‘The BLMcore K869A K870A construct was purified with the following modifications to remove a contaminating nuclease’.
>> This causes some concern, suggesting that the introduced mutations have actually affected protein fold (esp. K869A, see comments above) leading to non-specific hydrophobic interactions with other cellular proteins. An assessment of the stability of the protein fold is warranted, given this data, i.e. a thermal denaturation / thermofluor / DSF type of experiment.

We thank the reviewer for this comment and have carried out new experiments in response to this important question. To directly answer the reviewer’s question, the revised manuscript now includes DSF experiments on each protein tested in the paper. The BLM K869A K870A variant displayed a modest decrease in stability (WT BLM T_m_ is 46.3 °C and BLM K869A K870A T_m_ is 42.1 °C). Since our ATPase, EMSA, and helicase assays at temperatures below the T_m_, the moderate decrease in protein stability should not strongly impact the experiments tested in our manuscript. Additionally, we note that the BLM K869A K870A variant T_m_ was nearly identical to that of the BLM G1120R variant (42.6 °C) – since the BLM G1120R variant closely matched activity of the WT BLM protein, differences in activity for the BLM K869A K870A variant appear not be explained by altered stability. We have included our DSF data in our revised manuscript (Figure S2 and Figure 5) and have included data on the effects of ADP and ATPγS addition on stability of each variant (each ligand stabilized all BLM proteins in DSF, consistent with binding to properly folded proteins).

Page 11 — Molecular dynamic simulations
>> It is not fully clear how these simulations contribute to the overall conclusions of the paper. To a non-expert, the data presented in Figure 5 is generally not interpretable / easily understood. Also, to the non-trained eye the data presented in the lower section of panel A looks identical, with the overlay of the structure just serving to obfuscate the presented data. The authors also make several statements in the manuscript along the lines of “changes were not observed in our biochemical assays” when referring to predictions made by the simulation, again questioning its value / validitly.

Our previously published cellular assays in yeast (PMID: 23129629) and human cells (PMID: 26788541) show that the P868L mutation (partially) impairs BLM function. It is an interesting mutation because it is not located at or near the catalytic cleft of the BLM helicase core. Therefore, in this study, we carried out MD simulations to gain some insight into possible molecular effects of the P868L mutation on the BLM-DNA complex. Our simulations reveal two key effects of P868L mutation: (i) it increases BLM-DNA distances (Fig 6A) and (ii) it changes the relative orientations of the ZN, D2, RecAD1 domains (Fig 6B). We have now revised the text to make this point clearer, including the text in the figure 6 caption. Additionally, we now represent the structure in Figure 6A using a different angle, which shows more clearly the effect of P868L mutation on BLM-DNA distance. Finally, we do realize (in retrospect) that the statement “changes were not observed in our biochemical assays” was confusing. We have revised this sentence to clarify that the in vitro biochemical experiments we used may not have been sensitive enough to pick up the effect of P868L on BLM activity.

Page 13 — ‘BLMcore K869A K870A was found to have a modestly different maximum ATPase rate compared to WT BLMcore (P value = 0.0348)’.
>>Why is this change ascribed more weight/significance that that between WT BLMcore and BLMcore P868L (P value = 0.0116)? Also, a 35% difference between the maximum ATPase rates of WT BLMcore and BLMcore P868L is not a “relatively small difference”. This section needs revisiting and conclusions reconsidered.

We thank the reviewers for their comment. Since maximum ATPase rate scales with protein concentration in the assay, changes of less than one-fold are very difficult to interpret since they may be related to slight differences in measurements of the concentration of proteins used in assays. In contrast, K_DNA_ is not dependent on protein concentration. Because of this, we tried to emphasize differences between K_DNA_ instead of maximum ATPase rate. We do not try to draw any major conclusions from the changes in maximum ATPase rate among the BLM variants in this manuscript and make our conclusions from the K_DNA_ changes instead. We have noted this rationale in our manuscript.

Page 14 — Table 2, ATPase Data Summary
>>It is not clear why BLMcore K869A K870A has no measurable basal rate of ATP hydrolysis. This again points to potential protein fold issues (see points raised above).

As noted above, we have now measured the thermal stability for each BLM protein tested in the paper. Our DSF experiments indicate that the T_m_ BLM_core_ K869A K870A is 42.1 °C and that its stability increases in the presence of ADP or ATPγS, so the protein is predicted to be well folded in our ATPase assay, which is carried out at 25 °C. The DSF experiments inspired by the reviewer’s prior comment were very helpful in addressing this question.

Page 18 — Lines 421 to 422 / Figure 4
>> The data presented in Figure 4c indicates that BLMcore K869A K870A is only able to achieve unwinding of ~65% of the supplied substrate (as judged by the plateau region) of the plotted data. This again indicating problems with fold, i.e. not all the supplied protein is active / less stable or more likely is not fully soluble at higher concentration.

As noted above, we have now measured the thermal stability for each BLM protein tested in the paper, supporting a model in which decreased helicase activity is not due to protein instability in the variant.

Discussion
>> This part of the manuscript is unfortunately rather weak and is generally overly speculative in nature, and provides very little insight into the effects of either the P868L or G1220R polymorphisms on the in vivo functions of BLM helicase; e.g. “While BLM G1220R functions similarly to WT BLM in our assays, the G1220R substitution could cause a decrease in BLM binding affinity for HJs.” — so why not test this hypothesis?

We thank the reviewer for this comment. We attempted to address this issue by measuring HJ binding using our fluorescence-based EMSA and unwinding using our helicase assay with a synthetic HJ structure. Unfortunately, neither assay led to conclusive results. Several factors may have led to failure in this line of experimentation. For example, BLM activity on HJ substrates can be influenced by domains outside of the catalytic core and RPA has a strong influence on BLM unwinding of some HJ structures.  Since we cannot rigorously test the idea originally posed in our Discussion, we have removed it from the revised manuscript.

MINOR POINTS

Page 5, Line 109 —
>> Do either the P868L or G1220R polymorphisms introduce a rare codon, or are positioned at a known splice site, intron/exon boundary? What are the predicted effects on fold of either amino acid change (as predicted by ‘Site Directed Mutator” similar web-based analysis server”).

The G1120R and P868L polymorphisms do not introduce a rare codon according to the gnomAD database nor are they at known splice sites. We attempted to use Site Directed Mutator to look at the predicted impacts of these substitutions on protein structure and function, but on multiple days, different web browsers, and different computers, we were not able to access the server webpage.

Figure 1
>> provides insufficient molecular detail and should ideally include indications of known hydrogen-bond / polar / hydrophobic / stacking interactions. Side chain representations for the amino acids flanking Gly1220 should also be shown (GSKS motif).

We thank the reviewers for this comment. While it could be useful to display Figure 1 in this way, the main purpose of this figure was to orient the reader to what domains and what larger structural features the G1120R and P868L mutations were embedded within. We wanted to keep the figure as simple as possible so that the main focus is on the residues targeted in this study.

Figure 4, panel C
>> Please plot all data on axes with the same ranges.

While we would like to plot all of these data on the same axes, we used different concentrations of each BLM variant and therefore cannot have all BLM variants on the same axis.

Standard Error of the Mean
>> The authors need to clarify if the error bars represent standard error of the mean (SEM) or in fact 1 standard deviation (1 SD). SEM requires the mean from more than one experiment for calculations; do each of the indicated ‘three replicates’ contain more than one measurement?

We have included the standard error of the mean (SEM). The defined SEM is the standard deviation (SD) divided by the square root of N. It might be more appropriate to define the term as “SE” in the manuscript. We have included information in the manuscript about how this measurement is calculated in Prism.

Reviewer #2: Manuscript of Cuenty et is entitled as “Biochemical Properties of Naturally Occurring Human Bloom Helicase Variants”. This study is focussed on to assess the biochemical characterization of two non-BS BLM mutations e.g., P868L and G1120R. Previously, others have shown that mutation of these two aa led to defective DSB repair and enhanced sister chromatic exchange (22). In this manuscript, authors have tried assess the ATPase, DNA binding and helicase activity of these two mutations. Beside, they have assessed the effect of lysine rich surrounding near P868L. Overall, the current investigation is advancing the biochemical characterization of different heterozygous mutations in BLM and may have some relevance to understand the defective functions of P868L and G1120R BLM in genome instability at cellular level. Manuscript is well written but lacks novelty in term of correlating specific biochemical properties with the defects at cellular level. I have following concerns, which may be addressed to enhance the quality of the manuscript

1. Line 108-111: “……..Interestingly, two substitutions, P868L and G1120R, led to a partial loss in function, demonstrated by increased sensitivity to hydroxyurea compared to wild-type (WT) BLM. These mutations also cause a 4-5 fold increase in SCEs and increased sensitivity to hydroxyurea in human cells (22).” This statement is not fully correct. As per the citation (22), mutations at P868L and G1120R show no or moderate HU sensitivity. Authors need to correct this.

We thank the reviewer for pointing this out. We have corrected this statement in the manuscript to reflect that there was a moderate increase in HU sensitivity but only at low concentrations of HU. We have also included information about the increased sensitivity to CPT.

2. “……..There are over 1400 BLM mutations that exist in the human population, with 62 encoding for pathogenic mutations, 118 mutations considered benign, and the rest of uncertain or conflicting human health relevance (20).” Among so many pathogenic and non-characterized mutations, how authors have chosen to study P868 and G1120 mutations. The rationale of choosing these specific non-BS BLM mutations is not very convincing and needs proper justifications in the introduction of manuscript. For example, what mutations are known in helicase and RQC domain and why these two mutations, among all, were chosen for the current study

We thank the reviewer for this comment. Our rationale for choosing to study the P868 and G1120 mutations was because it had been previously shown that these mutations can cause a moderate increase in sister chromatid exchanges in tissue culture but did not cause Bloom syndrome. We felt that investigating these mutations would be beneficial since we already knew that they have some moderate impacts on BLM function in cells but these mutations are otherwise uncharacterized in their function as helicases and ATPases.

3. Line 295-297: “While BLMcore 295 P868L had an apparent increase in maximum ATPase rate compared to WT BLM (P value = 0.0116), there is a relatively small difference (35%) between the WT BLMcore and BLMcore P868L maximum ATPase rates (Table 2).” 35% difference is significant and not small.

We thank the reviewer for their comment. A similar comment was made by the first reviewer and our reply is added here as well. Since maximum ATPase rate scales with protein concentration in the assay, changes of less than one-fold are very difficult to interpret since they may be related to slight differences in measurements of the concentration of proteins used in assays. In contrast, K_DNA_ is not dependent on protein concentration. Because of this, we tried to emphasize differences between K_DNA_ instead of maximum ATPase rate. We do not try to draw any major conclusions from the changes in maximum ATPase rate among the BLM variants in this manuscript and make our conclusions from the K_DNA_ changes instead. We have noted this rationale in our manuscript.

4. Authors have done MD simulations of BLMcore P868L, which show some interesting observation that P868L has no major impact on DNA-D2/ZN distance but it enhances DNA-WH distance, which I personally feel has some effect on ATPase activity of P868L.

We thank the reviewers for this comment. We would anticipate that with the MD simulation changes between DNA and WH distance, that this would have an impact on ATPase activity. This could be related to the increased ATPase rate observed with BLM_core_ P868L, but since maximum ATPase rate is related to protein concentration, it is difficult to ascribe these changes to the P868L substitution. Additionally, it is possible that with different DNA substrates (such as duplex DNA) that there would be a larger difference in ATPase rates and K_DNA_ values with the BLM_core_ P868L mutation, but it would be more difficult to interpret these results since BLM would be able to unwind the substrate during the experiment.

5. Since these mutations have no major impact on DNA binding and helicase activity but shows significant effect on SCE and CPT induced replication DSB repair (22), it seems these residues may have some role with reference to different DNA specific structures. Authors should assess the DNA binding activity of P868 and G1120 for replication fork and Holliday junction substrates.

We thank the reviewer for their comment. A similar comment was made by the first reviewer and our reply is added here as well. To address this issue, we have tried to assess HJ binding using the same fluorescent based EMSAs that we used for the paper with HJ structure oligos, and unfortunately these assays had inconclusive results. We are not sure why this did not work. We also tried to assess HJ unwinding by BLM_core_ and had inconclusive results. Since we cannot rigorously test this idea, we have removed it from the discussion.

5. Why authors have not done MD simulation for other mutations (G1220R and K869A-K870A)?

We carried out MD simulations on only the BLM_core_ P868L variant because of how prevalent the P868L polymorphism is in the human population. Because of the time consuming and computing heavy nature of MD simulations, we did not carry out simulations on the other variants, but this will be an interesting target for future studies.
